# Sinonasal inverted papilloma associated with squamous cell carcinoma

**DOI:** 10.2478/v10019-011-0033-4

**Published:** 2011-10-08

**Authors:** Jasna But-Hadzic, Klemen Jenko, Mario Poljak, Bostjan J Kocjan, Nina Gale, Primoz Strojan

**Affiliations:** 1 Department of Radiation Oncology, Institute of Oncology Ljubljana, Ljubljana, Slovenia; 2 University Department of Otorhinolaryngology and Cervicofacial Surgery, University Clinical Centre Ljubljana, Ljubljana, Slovenia; 3 Institute of Microbiology and Immunology, Medical Faculty, University of Ljubljana, Ljubljana, Slovenia; 4 Institute of Pathology, Medical Faculty University of Ljubljana, Ljubljana, Slovenia

**Keywords:** inverted papilloma, squamous cell carcinoma, radiotherapy, human papillomavirus infection, outcome

## Abstract

**Background:**

The aims of the study were to review single-institution experiences with sinonasal inverted papilloma associated with squamous cell carcinoma (IP/SCC), to analyze the presence of human papillomavirus (HPV) and to evaluate the role of radiotherapy.

**Patients and methods:**

Five patients with IP/SCC were identified in the prospective institutional databases (1995–2005) and HPV status was determined in all five tumors.

**Results:**

Four out of five patients had T3-4 tumors; no nodal involvement was seen in any of them. Four patients had curative surgery, supplemented in three of them with radiotherapy. Debulking surgery was performed in the patient with a non-resectable tumor followed by radical radiotherapy. Tumor was controlled locally in three patients at 8, 46 and 58 months post-surgery. Local failure occurred in two patients: after endoscopic resection of a T1 tumor (the recurrent tumor was successfully salvaged with additional surgery) and in a patient with an inoperable tumor. No regional or distant metastases occurred. HPV status was determined in all five tumors and three of them were found positive for HPV type 11.

**Conclusions:**

In operable sinonasal IP/SCC, upfront surgery and postoperative radiotherapy to the tumor bed with dose levels comparable to those used for invasive SCC are recommended. For non-resectable disease, radical radiotherapy to a dose of 66–70 Gy could be of benefit.

## Introduction

Inverted papilloma (IP, one of three types of Schneiderian papilloma) of the nasal cavity and paranasal sinuses is a benign epithelial tumor of unknown etiology, first described by Ward in 1854.^1^ IP represents 0.5–4% of all sinonasal tumor and arises from the mucosa of the lateral wall of the nasal cavity, almost always unilaterally. IP is best characterized by the male-to-female ratio of 3:1 with the peak incidence between 5^th^ and 6^th^ decade of life, destructive pattern of local growth, tendency to recur and, occasionally, associated malignancy.^2^,^3^ The most common presenting symptom is unilateral nasal obstruction; the duration of symptoms is variable, with an average of >5 years but even up to >45 years.^2^–^4^

The frequency of carcinoma in patients with sinonasal IP is around 11%. In two thirds of cases, carcinoma occurs synchronously with IP, but in some patients carcinoma develops at a later time, after previous resection of IP (metachronous carcinoma). The associated malignancy is predominantly squamous cell carcinoma (SSC), which may arise within the papilloma or is merely associated with a histologically bland IP.^2^ This group of patients is characterized by older age and male preponderance compared with those without associated malignancy.^2^,^3^ An etiological role for human papillomavirus (HPV) and the mutation of the p53 tumor suppressor gene in malignant transformation of IP has been suggested.^5^–^7^

Literature reports on IP associated with SCC (IP/SCC) are scarce, describing only small cohorts of patents treated with varying degree of success. In the present report, we describe a group of five patients treated for IP/SCC. The presence of HPV was analyzed and the role of adjuvant radiotherapy was discussed.

## Patients and methods

**FIGURE 1:**
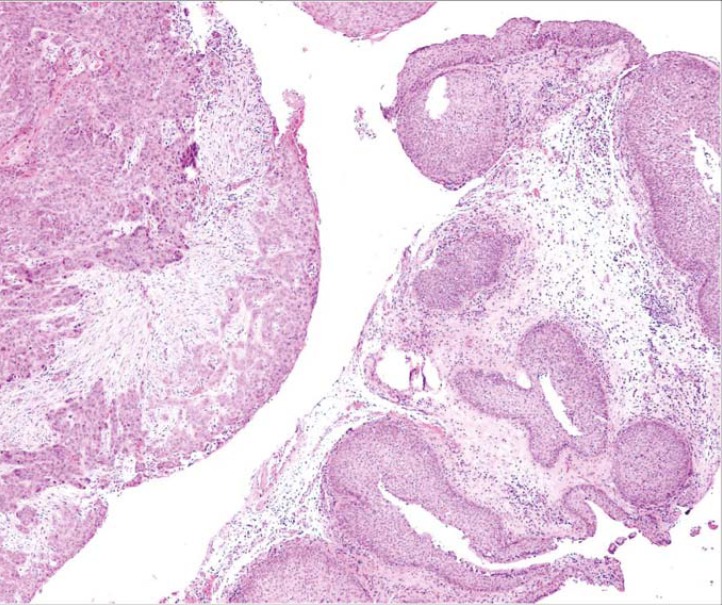
Inverted squamous papillomas are seen on the right side, synchronous moderately differentiated non-keratinizing invasive squamous cell carcinoma on the left side.

The databases of the University Department of Otorhinolaryngology and Cervicofacial Surgery, Clinical Center Ljubljana and the Institute of Pathology, Medical Faculty University of Ljubljana for the years 1995–2005 were used for identification of patients with a diagnosis of IP of the nasal cavity and paranasal sinuses. Out of 89 patients with this diagnosis, 5 patients (5.62%) were found to have IP/SCC. Pathology specimens of all 5 patients were re-examined by an experienced head and neck pathologist (N.G.) (Figure 1) and the medical records of identified patients were reviewed for clinical characteristics, treatment and outcome.

### Detection of HPV DNA

Tissue processing, DNA extraction and quantification as well as HPV detection and genotyping were described elsewhere.^7^ Briefly, total DNA was extracted from two 10-μm sections of paraffin blocks using QIAamp DNA FFPE Tissue Kit (Qiagen, Hilden, Germany), following the manufacturer’s instructions. For detection of HPV, PCR amplification was performed on all samples using HotStarTaq® *Plus* DNA Polymerase kit (Qiagen) and consensus GP5+ and GP6+ primers targeting approximately 150-bp fragments of alpha-HPV L1 gene. The PCR products that appeared as visible bands of the expected size were purified by QIAquick PCR Purification Kit (Qiagen) and sequenced directly using BigDye Terminator v1.1 Cycle Sequencing Kit (PE Applied Biosystems, Foster City, USA) with GP5+/GP6+ primers. A comparison of the HPV-DNA sequences obtained with those of officially designated alpha-HPV genotypes was carried out using the Blast server.^8^ Results from the Blast comparison software were confirmed additionally by pairwise alignment using the sequence of interest and full L1 gene of a reference HPV genotype.^9^

## Results

### Patients and tumors

Detailed information on the clinical characteristic of patients and their tumors, treatment and outcome is given in Table 1. There were four males and one female, from 45 to 77 years old (median 73 years). Unilateral nasal obstruction was the most frequent presenting symptom reported by four patients. The duration of symptoms before a diagnosis of IP with or without associated SCC was confirmed ranged from 2–6 months (median: 5 months).

In all but one patient, the disease extended from the nasal cavity to neighboring structures and no nodal or distant metastases were presented at the time of diagnosis in any of them. According to the UICC TNM staging system (7^th^ edition, 2009) for malignant tumors of the nasal cavity and ethmoid sinus, four patients had locally advanced T3-4 disease, whereas using the Krouse staging system,^10^ all tumors were categorized as stage T4 (*i.e.* due to associated malignancy).

The presence of SCC was confirmed by histopathological examination of resected specimen (*i.e.* after a biopsy proved negative for the presence of SCC) in two patients (pts. 2 and 5). The other three patients had metachronous SCC found in recurrent IP at 3, 10 and 24 years after first surgery (in pt. 4 at the time of resection of the sixth recurrence of IP). An invasive SCC component was found in all cases.

### Treatment

Four patients were operated on with curative intent. The surgical technique was dictated by the extent of the disease: a transnasal endoscopic technique and an external approach were used in two patients each. Surgery was declared as radical, without microscopic residual disease left behind, in all four patients, and all but one were irradiated postoperatively. The patient with extensive local disease, extending to the right ethmoid complex, sphenoid, pterygopalatinal fossa and orbita, had only debulking surgery followed by radiotherapy as a definitive treatment. None of the patients received systemic chemotherapy.

Postoperatively, continuous-course radiotherapy of 5 fractions per week was delivered in three patients, using megavoltage 5-MV or 6-MV linear accelerator photon beams, to a total tumor dose of 60 Gy. In two patients, the daily dose was 2 Gy and in one patient 2.5 Gy per fraction. The patient with gross residual disease after surgical debulking received definitive radiotherapy using megavoltage Co-60 photons in daily fractions of 2 Gy to a total dose of 70 Gy. Two-dimensional computer-based planning was used to cover the postoperative tumor bed or gross tumor volume including sinuses at risk for containing microscopic disease with a ≥95% isodose curve. A conventional three-field technique employing a heavily weighted anterior field and two opposed lateral wedged fields to achieve dose homogeneity in the range of ±5% inside the treated volume, head holders with thermoplastic casts and individual shielding blocks were used. When appropriate, optic structures were shielded after a dose of 54 Gy. The ipsilateral regional lymphatics (regions II–V) were irradiated postoperatively in one patient (pt. 5), through an anterior field and with 2.5 daily fractions to a total dose of 40 Gy, whereas in another patient (pt. 2), region II was covered ipsilaterally to a dose of 50 Gy in 2-Gy daily fractions. In both patients, indication for neck irradiation was extensive local disease.

#### Outcome

After diagnosis of IP/SCC, two patients were alive at 58 and 70 months. In the patient treated solely with endoscopic resection (patient 1), isolated local recurrence, histologically confirmed as IP/SCC, developed on the nasal septum 8 moths later; an endoscopic salvage procedure resulted in a permanent local control of 62 months. Two patients died of disease-unrelated causes without disease reappearance at 8 and 46 months after diagnosis of IP/SCC; at the time of death, they were 74 and 81 years old, respectively. The patient who underwent definitive radiotherapy died due to progression of residual disease at 14 months from biopsy.

No severe or unexpected complications of treatment were documented during therapy and none of the patients had severe late therapy-related sequelae that would demand surgical intervention or hospitalization.

#### Human papillomavirus

Three out of five tumors analyzed for the presence of HPV were found positive, with type 11 being present in all positive cases.

## Discussion

In IP, whether or not associated with SCC, complete surgical removal of the tumor is advocated as the treatment of choice. Endoscopic treatment is preferred, whereas for lesions less accessible endoscopically, or in those with peripheral extension, open surgery is indicated.^11^,^12^ When complete resection is not possible, or for tumors with associated malignancy, radiotherapy is recommended as an adjunct to surgery.^13^,^14^

While the experiences with IP are extensive^4^,^10^–^12^, series describing IP/SCC are limited and feature a limited number of cases. Tanvetyanon *et al.*^15^ collected survival data of 76 patients from his series and ten additional series published during the last 30 years, covering a recruitment period of almost six decades.^4^,^13^–^22^ The corresponding pooled median overall survival was 126 months with 3-year survival estimate of 63%, which is in the range reported for invasive SCC of the nasal cavity and paranasal sinuses^23^ but much lower compared to figures appearing in IP series.^4^,^11^,^12^

Recommendations for the use of radiotherapy are based on clinical observations rather than scientific analyses. More than three quarters of 76 patients from pooled group reported by Tanvetyanon *et al.* had one or another form (*i.e.* pre- or postoperative, definitive, palliative) of irradiation but no analysis on the value of radiotherapy versus surgery alone was carried out in their study.^15^ After detailed review of the studies analyzed in the paper mentioned above, we conclude that the low quality of information on the tumor extent and treatment, including completeness of surgical resection, in some publications must be the reason.

According to Hug *et al.*^13^ and Gomez *et al.*^14^, the probability of regional or systemic dissemination of IP/SCC is low. Consequently, they proposed elective irradiation of regional lymphatics only in patients with extensive involvement of the nasopharynx or clinically or radiologically apparent neck metastases. Our limited experience corroborates their recommendations.

Because of generally accepted opinion that radiotherapy should not be used as an adjunct to surgery in bland IP, in IP/SCC cases the choice of irradiation dose level is usually dictated by the recommendations for invasive SCC. This is obviously the case when series with sufficiently described radiotherapy details are reviewed.^14^,^15^,^17^,^20^ In 19 pre- or postoperatively irradiated patients, radiotherapy doses ranged from 45 Gy to 70.4 Gy (median 60 Gy); all but four patients received 58.4–66.8 Gy. Furthermore, in a group of 13 patients with IP/SCC reported by Hugh *et al.*^13^ only one local failure occurred after gross total resection and adjuvant hyperfractionated or conventionally fractionated radiotherapy to a mean dose of 59 Gy or 60 Gy, respectively. Following subtotal resection, one out of three tumors failed locally after hyperfractionated irradiation to a mean dose of 66 Gy.^13^ In the present series, all three tumors postoperatively irradiated were locally controlled at 8, 46 and 58 months post-diagnosis; an irradiation dose of 60 Gy was adjusted to the tumor stage (T3-T4) and the presence of the SCC component.

Experience with chemotherapy in IP/SCC is very scarce. In the majority of cases, chemotherapy was aimed at palliating symptoms of unresectable disease, locally or at distant sites,^15^,^20^ or has exceptionally been used for reducing tumor size before surgery^15^,^21^, or in a postoperative setting, usually with irradiation.^15^,^19^–^21^ The chemotherapeutics used were platinum compounds, 5-fluorouracil, paclitaxel, etoposide, and methotrexate.^15^,^21^ No conclusions could be made on the effectiveness of chemotherapy in SCC/IP; however, none of the three patients with distant metastases reported by Tanvetyanon *et al.*^15^ responded to any of the chemotherapy regimens used.

Presence of HPV type 11 was confirmed in three out of five tumors from the present series. Similarly, Cheung *et al.*^5^ demonstrated the presence of HPV in four out of seven IP/SCC cases, one of them being type 11 (typing was not done in other cases because of inadequate HPV DNA content). Simultaneously decreased expression of p16 found in above cited and other studies indicates that the role of HPV in the oncogenesis of IP/SCC differs from that in cervical SCC. It seems that HPV infection occurs as an early event in the multistep process of malignant transformation from IP to SCC.^5^,^6^,^24^ However, others suggested that HPV infection may represent incidental colonization rather than being and important etiological factor.^7^

Rather high prevalence of HPV infection in IP but also IP/SCC specimens poses clinically relevant question on the potential prognostic significance of HPV status. Patients with HPV-positive SCCs of the head and neck, oropharynx in particular, have superior outcome, attributed to enhanced radiation and chemo-sensitivity due to an intact apoptotic mechanism in response to radiation and chemotherapy.^25^,^26^ Because in oropharyngeal SCCs HPV types 16 and 18 rather than 11 are usually found, the question of radio/chemo-sensitivity of HPV-positive IPs and IP/SCCs at this point remains to be elucidated. However, unexpectedly favorable responses after radiotherapy have also been reported in extensive IPs and SCC/IPs. Myers *et al*.^17^ described a case of IP/SCC destroying the bony walls of the antrum with orbital invasion; no residual IP or SCC was found in the surgical specimen after 60 Gy of preoperative radiotherapy. A similar experience with preoperative irradiation was reported by Gomez *et al*.^14^, whereas in the patient with non-resectable IP with bilateral involvement of the nasal cavity and paranasal sinuses, radiotherapy alone with 65 Gy was deemed curative (no recurrence at 7 years). The authors draw attention to the rather long interval after irradiation, from 3 to 6 months, for gross disease to disappear.^14^ Also, after surgical debulking of locally recurrent IP associated with carcinoma *in situ*, affecting the zygomatic area and with extension into the infratemporal fossa, Levendag *et al*.^27^ found irradiation to 64 Gy highly effective, resulting in complete regression of the lesion for almost one year.

## Conclusions

According to clinical experiences, combination of surgery and postoperative irradiation with radiotherapy dose levels in a range used for invasive SCC are recommended for operable IP/SCC. Elective neck irradiation should be considered only for patients with extensive nasopharyngeal involvement or apparent regional metastases. For non-resectable disease, radical radiotherapy to a dose of 66–70 Gy could be of benefit with potential for long-lasting remission or even cure.

## Figures and Tables

**TABLE 1:** Sinonasal inverted papilloma associated with squamous cell carcinoma: clinical and tumor characteristics, treatment and outcome

**Parameter**	**Patients**				

	**1**	**2**	**3**	**4**	**5**
Sex/Age (yrs.)	M/45	M/77	M/75	F/73	M/62
Presenting symptoms	Unilateral nasal obstruction	Unilateral nasal obstruction, nasal discharge, anosmia, pain	Nasal discharge	Unilateral nasal obstruction, headache, diplopia	Unilateral nasal obstruction blurred vision, headache, anopsia
Duration of symptoms	6 mos.	5 mos.	2 mos.	2 mos.	6 mos.
Extent of disease	Lt nasal cavity	Rt nasal cavity, nasopharynx	Rt nasal cavity, ethmoid, orbit	Rt nasal cavity, ethmoid, maxillary sinus, orbit	Rt nasal cavity, ethmoid, fossa pterygopalatina, maxillary sinus, sphenoid, orbit
TNM stage*	T1N0M0	T4bN0M0	T3N0M0	T4aN0M0	T4bN0M0
Histology at 1^st^ biopsy	P	IP	P	IP	IP
No. of recurrences	1	0	1	6	0
SCC type	M	S	M	M	S
Histpathological grade of SCC	n.s.	G II–III	G II–III	G II	n.s.
Surgery	Endoscopic resection; R0	Endoscopic resection; R0	Lateral rhinotomy; R0	Sublabial and external supraciliar approach; R0	Sublabial approach, explorative and debulking procedure; R2
HPV status	Positive (type 11)	Positive (type 11)	Positive (type 11)	Negative	Negative
Radiotherapy	Not irradiated	60 Gy, 30#, 5MV, 3 fields, continuous course; Tu site & Rt region II	60 Gy, 30#, 6MV, 3 fields, continuous course; Tu site	60Gy, 24#, 5MV, 3 fields, continuous course; Tu site & Rt neck	70 Gy, 35#, Co-60, 3 fields, continuous course; Tu site
Follow-Up	Local recurrence at 8 mos., NED 62 mos. after salvage surgery	DOC at 46 mos., no evidence of IP/SCC	NED at 58 mos.	DOC at 8 mos., no evidence of IP/SCC	DOD at 14 months, locally progressive disease

M – Male; F – Female; Rt – Right; Lt – Left; P – Papilloma; IP – Inverted papilloma; M – Metachronous; S – Synchronous; n.s. – Not specified; SCC – Squamous cell carcinoma; # – No. of fractions; Tu – Tumor. NED – No evidence of disease; DOD – Died of disease; DOC – Died of other cause.

* TNM clinical classification of malignant tumors of the nasal cavity and ethmoid sinuses.
